# (−)-Epigallocatechin-3-Gallate and Quercetin Inhibit Quiescin Sulfhydryl Oxidase 1 Secretion from Hepatocellular Carcinoma Cells

**DOI:** 10.3390/antiox14010106

**Published:** 2025-01-17

**Authors:** Lumin Yang, Yuying Fang, Yufeng He, Jinsong Zhang

**Affiliations:** State Key Laboratory of Tea Plant Biology and Utilization, School of Tea & Food Science, Joint Research Center for Food Nutrition and Health of IHM, Anhui Agricultural University, Hefei 230036, China; luminyang@stu.ahau.edu.cn (L.Y.); fangyy@stu.ahau.edu.cn (Y.F.); hyf@stu.ahau.edu.cn (Y.H.)

**Keywords:** QSOX1, hepatocellular carcinoma, EGCG, quercetin, sorafenib, secretion, synergy

## Abstract

Liver cancer is one of the most prevalent cancers worldwide. The first-line therapeutic drug sorafenib offers only a moderate improvement in patients’ conditions. Therefore, an approach to enhancing its therapeutic efficacy is urgently needed. It has been revealed that hepatocellular carcinoma (HCC) cells with heightened intracellular quiescin sulfhydryl oxidase 1 (QSOX1) exhibit increased sensitivity to sorafenib. QSOX1 is a secreted disulfide catalyst, and it is widely recognized that extracellular QSOX1 promotes the growth, invasion, and metastasis of cancer cells through its participation in the establishment of extracellular matrix. Inhibiting QSOX1 secretion can increase intracellular QSOX1 and decrease extracellular QSOX1. Such an approach would sensitize HCC cells to sorafenib but remains to be established. Since (−)-epigallocatechin-3-gallate (EGCG) has been demonstrated to be an effective inhibitor of α-fetal protein secretion from HCC cells, we screened QSOX1 secretion inhibition using polyphenolic compounds. We examined eight dietary polyphenols (EGCG, quercetin, fisetin, myricetin, caffeic acid, chlorogenic acid, resveratrol, and theaflavin) and found that EGCG and quercetin effectively inhibited QSOX1 secretion from human HCC cells (HepG2 or Huh7), resulting in high intracellular QSOX1 and low extracellular QSOX1. The combination of EGCG or quercetin, both of which change the cellular distribution of QSOX1, with sorafenib, which has no influence on the cellular distribution of QSOX1, exhibited multiple synergistic effects against the HCC cells, including the induction of apoptosis and inhibition of invasion and metastasis. In conclusion, our current results suggest that dietary EGCG and quercetin have the potential to be developed as adjuvants to sorafenib in the treatment of HCC by modulating the cellular distribution of QSOX1.

## 1. Introduction

Hepatocellular carcinoma (HCC) is one of the primary causes of cancer-related deaths globally [1]. In 2022, the global incidence and mortality rates for liver cancer were 8.6 and 7.4 per 100,000, respectively [2]. Sorafenib, a multikinase inhibitor, is the first-line therapeutic drug for advanced HCC [3]. However, only about 30% of HCC patients respond positively to sorafenib, and these patients typically develop resistance within 6 months [4]. The tumor microenvironment, epigenetic biological processes, regulated cell death, and transport processes have been identified to be associated with resistance to sorafenib [4]. Therefore, there is an urgent need for new therapeutic modalities that target pathways other than those targeted by sorafenib to extend the efficacy of sorafenib. To maintain the efficacy of sorafenib against HCC, its combination with immunotherapeutic drugs, molecular agents targeting EGFR or PI3K/AKT/mTOR signaling, or cytotoxic chemotherapeutic agents has been proposed [4]. Quiescin sulfhydryl oxidase 1 (QSOX1), a disulfide catalyst, oxidizes thiols to disulfide bonds [5]. It was recently found that a high expression of intracellular QSOX1 significantly increases the sensitivity of HCC cells to sorafenib [6]. Similarly, liver cancer patients with a high expression of QSOX1 in their tumors exhibited higher survival rates following postoperative sorafenib treatment [6]. In addition, HCC cells overexpressing QSOX1 were associated with reduced invasive and metastatic capabilities [7,8]. Moreover, a lower rate of recurrence was observed in liver cancer patients with high levels of QSOX1 in their tumors [7,8]. These results strongly indicate that intracellular QSOX1 may act as a tumor suppressor in HCC cells. Accordingly, a pharmacological approach increasing intracellular QSOX1 would be helpful for elevating the therapeutic efficacy of sorafenib.

QSOX1 is a secreted enzyme [9]. As such, a proteomic analysis found that serum QSOX1 was one of the most significantly increased proteins in liver cancer patients [10]. Moreover, serum dithiothreitol-oxidizing capacity (DOC; a surrogate test for QSOX1 activity) was significantly increased in patients with end-stage liver diseases, including HCC [11]. Accumulating evidence shows that extracellular QSOX1 increases the occurrence of malignant phenotypes of cancer cells. QSOX1 is essential for the formation of extracellular matrix (ECM) by catalyzing cysteine cross-linking [9]. It is well known that ECM is a key mediator between cancer cells and their surroundings, thereby promoting the proliferation, migration, or invasion of cancer cells [12,13,14]. Inhibiting extracellular QSOX1 activity by antibody blockade significantly disrupted the ECM structure, thereby reducing cancer cell adhesion, invasion, or migration in vitro [9,15,16] and suppressing tumor growth and metastasis in vivo [17]. Moreover, adding enzymatically active recombinant QSOX1 to the culture medium restored the invasive phenotype of tumor cells transduced with shQSOX1 [9,18]. These findings cumulatively suggest that extracellular QSOX1 is a tumor-promoting factor.

Given the dual role of QSOX1 in HCC’s progression, i.e., as an intracellular tumor suppressor and an extracellular tumor promoter, it is evident that restricting QSOX1’s secretion from HCC cells can increase intracellular QSOX1 and reduce extracellular QSOX1. Such an approach would synergize with sorafenib to obtain a much-improved therapeutic result in liver cancer patients. (−)-Epigallocatechin-3-gallate (EGCG), a primary polyphenol in green tea, has been shown to suppress the secretion of α-fetal protein (AFP) from HepG2 cells, leading to AFP aggregation in HepG2 cells and reduced AFP levels out of HepG2 cells [19]. Moreover, the anti-HCC properties of natural polyphenols have been extensively studied in recent years, and accumulating evidence suggests that many of these properties could affect a variety of molecular targets and signaling pathways of HCC cells [20,21,22]. In addition, synergistic effects of sorafenib and EGCG or quercetin (Que) against HCC in mice have been reported [23,24]. Therefore, in this study, we screen QSOX1 secretion inhibitors based on dietary polyphenols found in tea, coffee, fruits, and vegetables, including EGCG, Que, fisetin, myricetin, caffeic acid, chlorogenic acid, resveratrol, and theaflavin. Based on the eight polyphenols examined in HCC cells, we found that only EGCG and Que effectively induced the expected cellular distribution of QSOX1 (i.e., increased intracellular levels and reduced extracellular levels). EGCG or Que as an inhibitor of QSOX1 secretion from HCC cells could synergize with sorafenib, which does not significantly change the distribution of QSOX1 in HCC cells, to cause apoptosis and to inhibit invasion and metastasis.

## 2. Materials and Methods

### 2.1. Chemicals and Reagents

EGCG (purity > 99%) was sourced from Ebeikar Tea & Extracts Co., Ltd. (Hangzhou, China). Que (purity > 95%) was sourced from Sigma (St. Louis, MO, USA). Caffeic acid, chlorogenic acid, theaflavin, and resveratrol (all with purity >98%) were sourced from Weikeqi Biotechnology Co., Ltd. (Chengdu, China). Myricetin (purity > 97%) and fisetin (purity > 98%) were procured from Macklin (Shanghai, China). Sorafenib was sourced from Selleck (Houston, TX, USA). DMEM culture medium, trypsin, and penicillin–streptomycin solutions were acquired from HyClone (Logan, UT, USA). Fetal bovine serum was purchased from Gibco (Grand Island, NY, USA). A Transwell chamber and Matrigel were purchased from Corning (Cambridge, MA, USA). Trypan Blue was procured from Sigma (St. Louis, MO, USA). The primary antibody against QSOX1 (ab235444) was purchased from Abcam (Cambridge, UK). The primary antibody against β-actin (A5441) was purchased from Sigma (St. Louis, MO, USA). The primary antibodies against PARP (#9542), Caspase-8 (#9746), Cleaved Caspase-8 (#98134), Bax (#2772), and Bcl-2 (#2876), as well as anti-rabbit (#7074) and anti-mouse (#7076) secondary antibodies, were obtained from Cell Signaling Technology, Inc. (Boston, MA, USA). The Annexin V-FITC/propidium iodide (PI) apoptosis detection kit was sourced from MedChem Express (Monmouth Junction, NJ, USA). DTT, 5,5-dithiobis-2-nitrobenzoicacid (DTNB), HEPES, Tween-80, guanidine hydrochloride, and horseradish peroxidase were all acquired from Sigma (St. Louis, MO, USA). 3-(4,5-Dimethyl-2-thiazolyl)-2,5-diphenyltetrazolium bromide (MTT) was a product of Amresco LLC (Solon, OH, USA). Amplex UltraRed was purchased from Life Technologies (Carlsbad, CA, USA). All other chemicals employed were of the highest analytical grade available. Some healthy human or mouse serum samples employed in our previous research [11,25] were pooled together in this study to assess the potential influence of polyphenols on serum QSOX activity.

### 2.2. Cell Culture

HepG2 cells were obtained from the Stem Cell Bank of the Chinese Academy of Sciences in Shanghai, while Huh7 cells were provided by Anhui Medical University located in Hefei, China. Both HepG2 and Huh7 cells were cultured in DMEM supplemented with 10% fetal bovine serum, 100 μg/mL of streptomycin, and 100 U/mL of penicillin, and were incubated at 37 °C in a 5% CO_2_ environment.

### 2.3. MTT Assay

We employed the MTT staining assay developed by Momann [26] to determine cell viability. Briefly, cells were treated with the examined drugs for 48 h followed by removal of the medium and the addition of 200 µL of medium containing MTT (0.5 mg/mL) to each well. After a 4 h incubation, the medium was removed and 150 µL of dimethylsulfoxide was added. The optical density was measured at 490 nm using a microplate reader (Molecular Devices SpectraMax 190, Sunnyvale, CA, USA). The data are expressed as the percentage of live cells versus the control.

### 2.4. Trypan Blue Assay

The Trypan Blue assay was performed as described by Louis [27]. In brief, HepG2 or Huh7 cells were seeded in 24-well plates (1 × 10^5^ cells/well). The cells were treated with Que or theaflavin for 48 h followed by trypsinization, incubated with 0.4% trypan blue, and finally counted in a Neubauer chamber.

### 2.5. Cell Migration and Invasion Assay

To evaluate cell migration and repair, we employed the scratch assay technique. Cells were seeded in 6-well plates, and the cell layers was scraped with sterile pipette tips after adhesion. After 48 h of treatment with the drug, the relative migration ratio was calculated using the following formula: (width at 0 h–width at 48 h)/width at 0 h.

To evaluate the ability of cell migration and invasion, Transwell assays were conducted. The assays used 24-well Transwell chambers (8.0 μm pore size). For the invasion assay, the upper chamber was precoated with Matrigel at a dilution ratio of 1:20. Briefly, Huh7 cells were seeded in the upper chambers with 200 μL of serum-free medium. In the lower chamber, 600 μL of medium containing 20% FBS, with or without the drug, was added. After a 48 h culture, the cells were fixed with paraformaldehyde and stained with crystal violet. Finally, the cells were examined and counted in three random fields using the Axio Zoom.V16 stereomicroscope (Carl Zeiss AG, Göttingen, Germany) (70×).

### 2.6. Western Blot

Total cellular proteins were extracted using the Radio-Immune Precipitation Assay (RIPA) reagent, and the protein concentrations were determined using the BCA protein assay kit (Beyotime Biotechnology, Shanghai, China). Cellular protein extracts were mixed with loading buffer and boiled at 95 °C for 10 min. Subsequently, 20 μg of protein was separated via SDS-PAGE and transferred to a polyvinylidene difluoride (PVDF) membrane. For the extracellular QSOX1 protein assay, the cell culture supernatant was directly mixed with loading buffer and boiled at 95 °C for 10 min, and 10 μL of the mixture was sampled per lane, separated via SDS-PAGE, and transferred to PVDF membranes. A Western blot analysis was performed, as described previously [25], using specific primary and secondary antibodies. Proteins were detected using the ChemiDoc XRS + detection system (ECL, Bio-Rad, Hercules, CA, USA) and quantified through densitometry via the Quantity One^®^ Image Analyzer software version 4.6.2 program (Bio-Rad). Finally, the quantification data were normalized to the internal control.

### 2.7. High-Content Cell Imaging

High-content cell imaging was conducted following the protocols outlined by Kota et al. [28]. To visualize and quantify cellular responses, HepG2 cells were treated with drugs for 48 h. The medium was removed and the adherent cells were stained with 4′,6-diamidino-2-phenylindole (DAPI), Annexin V-FITC, and PI. Images were obtained using the Operetta high-content imaging system. The data were then analyzed with MetaXpress software version 6.1.1 (Molecular Devices, Sunnyvale, CA, USA).

### 2.8. QSOX and DOC Activity Assay

QSOX activity was measured at 25 °C following the protocol established by Israel et al. [29]. The DOC activity was determined according to the method described in our previous study [30].

### 2.9. Statistical Analysis

All statistical analyses were conducted using GraphPad Prism version 5. Data are presented as the mean ± SEM (standard error of mean). The significances between groups were assessed using Student’s *t* test, one-way ANOVA, or post hoc Dunnett’s or Tukey’s test, depending on the appropriateness of each method. The Bliss independence model, as described by Cao et al. [31], was used to analyze whether there were synergistic effects. Briefly, the presence of synergistic effects was determined by calculating ∆fa_xy_; if ∆fa_xy_ > 0, then the two drugs show synergistic effects. A *p* value of less than 0.05 was considered statistically significant.

## 3. Results

### 3.1. Only EGCG and Que Among Examined Polyphenols Alter Cellular Distribution of QSOX1

**Figure 1:**
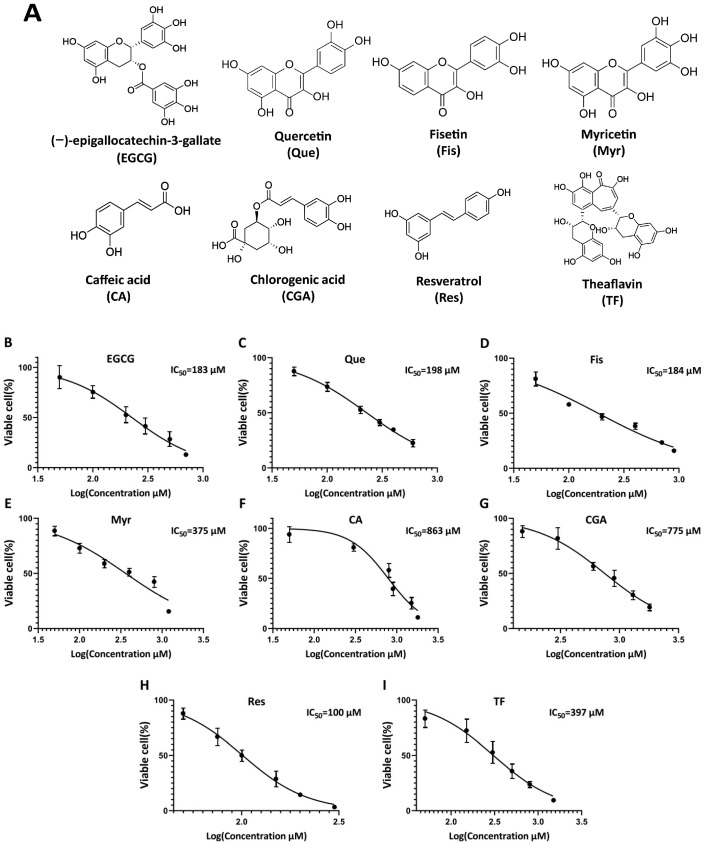
Influence of dietary polyphenols on cell viability in HepG2 cells. (**A**) Chemical structures of examined polyphenols. (**B**–**I**) Cell viability following a 48 h treatment. Data are presented as mean ± SEM (n = 6 in (**B**,**D**–**H**); n = 3 in (**C**,**I**)).

**Figure 2:**
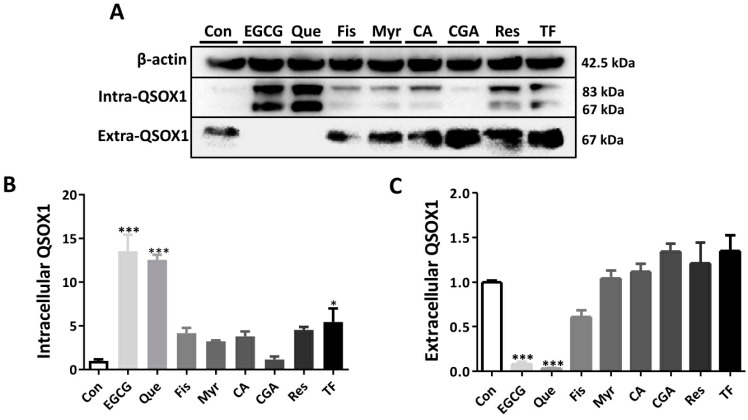
Influence of dietary polyphenols on intra- and extracellular QSOX1 in HepG2 cells. HepG2 cells were treated with polyphenol at IC50 for 48 h. (**A**) Western blot to confirm intra- and extracellular QSOX1 expression levels. (**B**) Quantification of intracellular QSOX1 levels in Western blot. (**C**) Quantification of extracellular QSOX1 levels in Western blot. Data are presented as mean ± SEM (n = 3). Compared to the control, * *p* < 0.05 and *** *p* < 0.001.

Zhang et al. [7] found that the expression of QSOX1 in HCC cell lines was negatively correlated with their metastatic ability, with the highest expression observed in HepG2 cells. Therefore, we first investigated the effects of different polyphenols on the intracellular and extracellular distribution of the QSOX1 protein in HepG2 cells. Eight dietary polyphenols, namely EGCG, Que, fisetin, myricetin, caffeic acid, chlorogenic acid, resveratrol, and theaflavin (Figure 1A), were used to treat HepG2 cells for 48 h to obtain their half maximal inhibitory concentration (IC50) values (Figure 1B). Then, the IC50 values of each polyphenol were used to observe their influence on cellular QSOX1 following a 48 h incubation. Only EGCG and Que significantly increased intracellular QSOX1 (containing two bands, QSOX1 long-chains and short-chains, respectively) and at the same time significantly decreased extracellular QSOX1 (Figure 2). Thus, EGCG and Que were subsequently used for characterizing their influence on cellular QSOX1 under non-cytotoxic conditions.

### 3.2. EGCG and Que Under Non-Cytotoxic Conditions Inhibit QSOX1 Secretion

**Figure 3:**
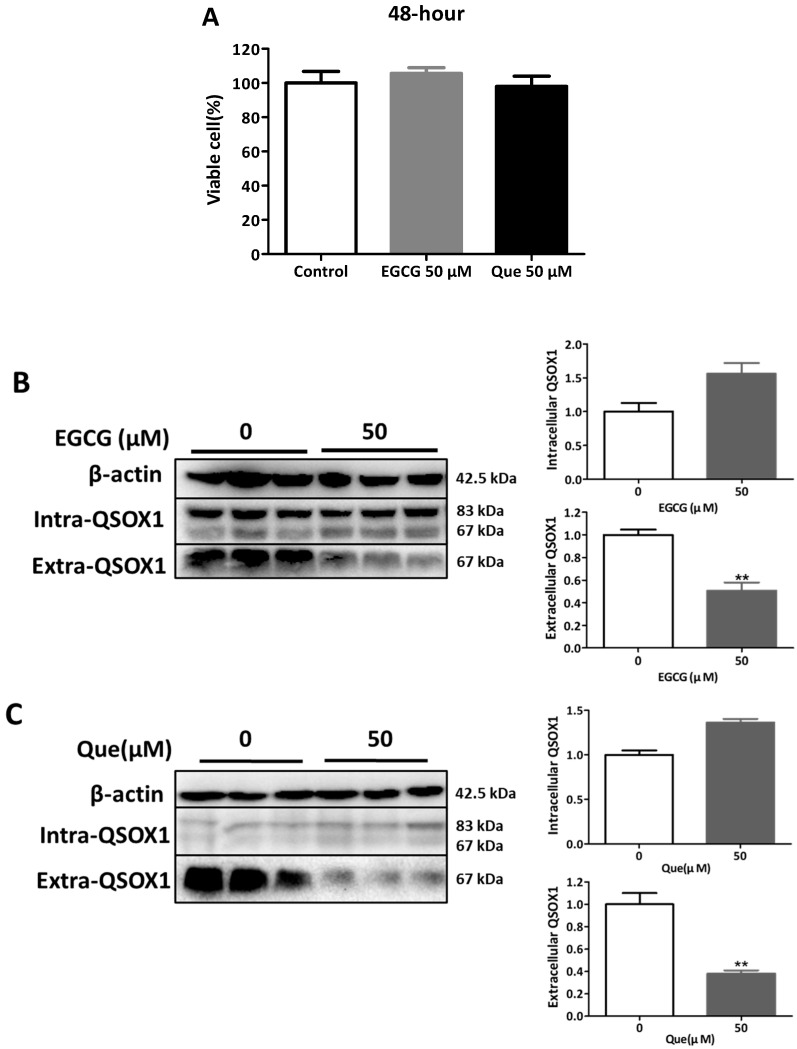
Influence of EGCG and Que on intra- and extracellular QSOX1 of HepG2 cells in non-cytotoxic conditions. HepG2 cells were treated with EGCG (50 μM) or Que (50 μM) for 48 h. (**A**) Cell viability; (**B**) QSOX1 protein following treatment with EGCG; (**C**) QSOX1 protein following treatment with Que. Data are presented as mean ± SEM (n = 6 in (**A**); n = 3 in (**B**,**C**)). Compared to the control, ** *p* < 0.01.

**Figure 4:**
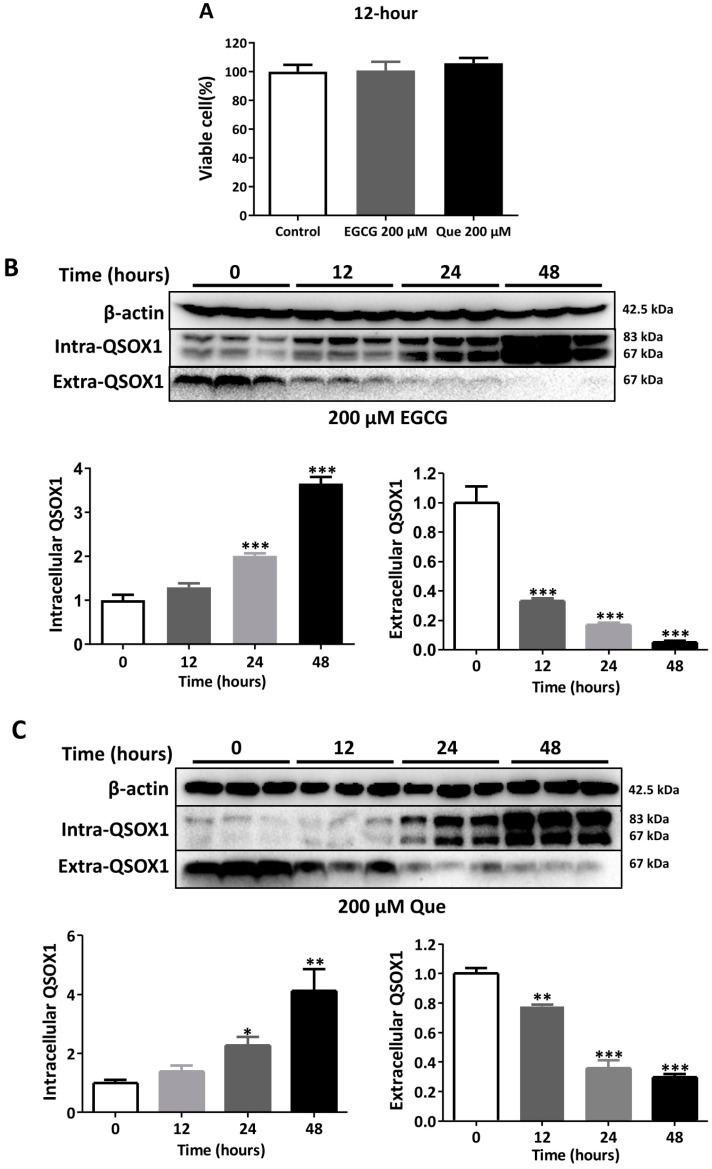
Time course of alterations in QSOX1 subjected to EGCG or Que treatment in HepG2 cells. (**A**) Cell viability following a 12 h treatment of 200 μM EGCG or Que; (**B**) HepG2 cells treated with 200 μM EGCG for 0, 12, 24, or 48 h; (**C**) HepG2 cells treated with 200 μM Que for 0, 12, 24, or 48 h. Data are presented as mean ± SEM (n = 6 in (**A**); n = 3 in (**B**,**C**)). Compared to the control, * *p* < 0.05, ** *p* < 0.01, and *** *p* < 0.001.

**Figure 5:**
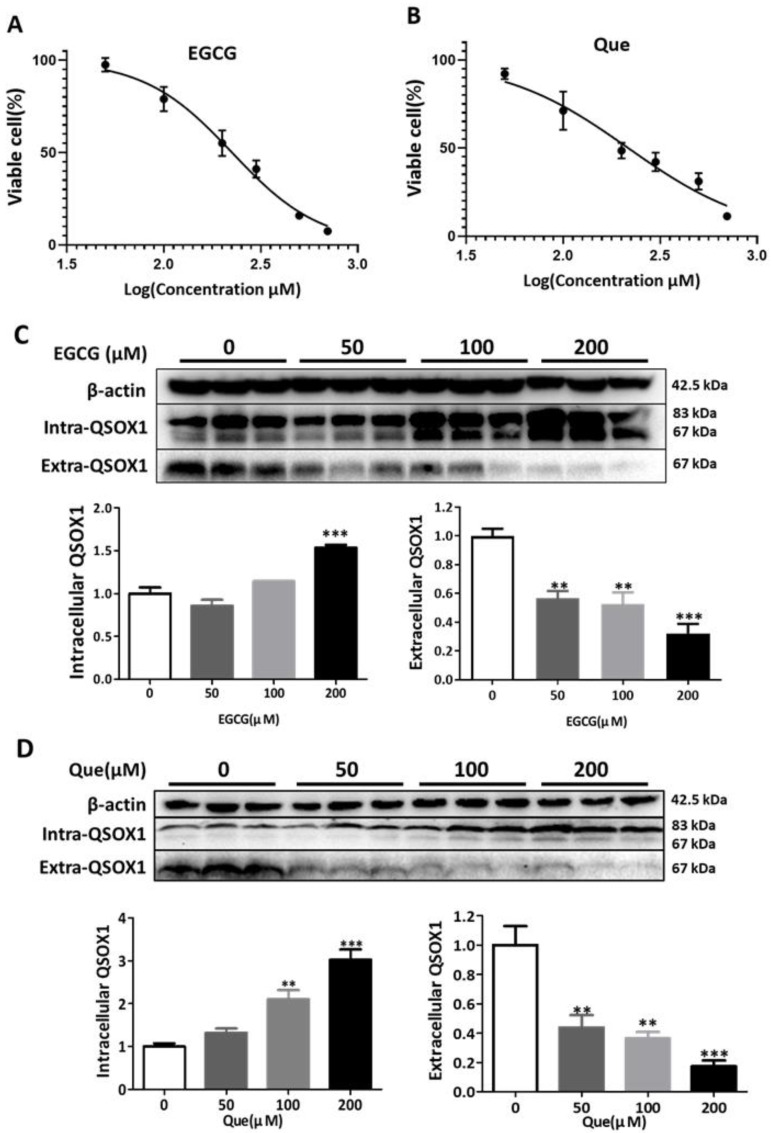
Influence of EGCG and Que on intra- and extracellular QSOX1 of Huh7 cells in non-cytotoxic conditions. (**A**,**B**) Cell viability following a 48 h treatment; (**C**) Huh7 cells treated with 0, 50, 100, or 200 μM EGCG for 48 h; (**D**) Huh7 cells treated with 0, 50, 100, or 200 μM Que for 48 h. Data are presented as mean ± SEM (n = 6 in (**A**); 3 in (**B**–**D**)). Compared to the control, ** *p* < 0.01 and *** *p* < 0.001.

**Figure 6:**
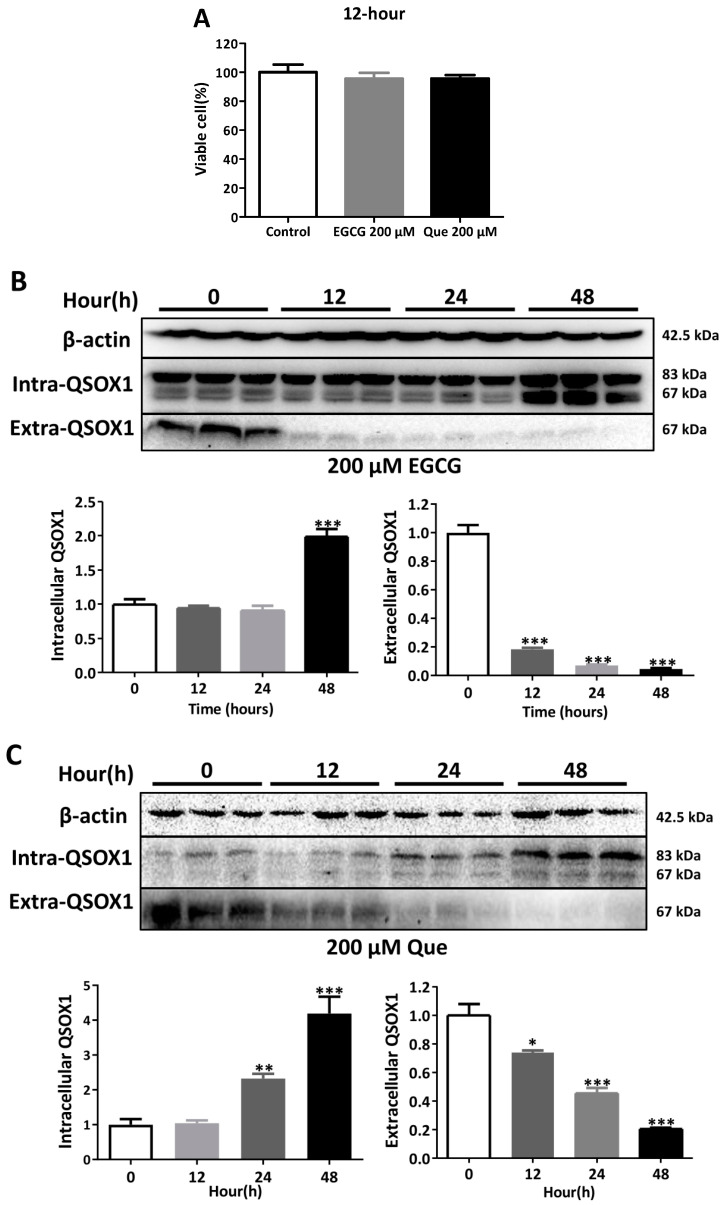
Time course of QSOX1 alterations subjected to EGCG or Que treatment in Huh7 cells. (**A**) Cell viability after treatment with EGCG or Que for 12 h; (**B**) Huh7 cells treated with 200 μM EGCG for 0, 12, 24, or 48 h; (**C**) Huh7 cells treated with 200 μM Que for 0, 12, 24, or 48 h. Data are presented as mean ± SEM (n = 6 in (**A**); n = 3 in (**B**,**C**)). Compared to the control, * *p* < 0.05, ** *p* < 0.01, and *** *p* < 0.001.

It was found that 50 μM EGCG significantly inhibited the secretion of AFP in HepG2 cells and induced the intracellular aggregation of AFP [19]. In our study, treatments with 50 μM EGCG or Que for 48 h did not affect the viability of HepG2 cells (Figure 3A) but did significantly inhibit extracellular QSOX1 (Figure 3B,C). Treatments with 200 μM EGCG or Que for 12 h did not affect the cell viability of HepG2 cells (Figure 4A) but did significantly inhibit extracellular QSOX1 (Figure 4B,C). Moreover, treatments with 50 μM EGCG or Que for 48 h and treatment with 200 μM EGCG or Que for 12 h significantly inhibited extracellular QSOX1, although none of them affected the viability of Huh7 cells (Figure 5 and Figure 6). These results derived from non-cytotoxic conditions suggest that EGCG and Que are able to reduce the secretion of QSOX1 from human HCC cells. As a result of secretion inhibition seen at 12 h, we observed that intracellular QSOX1 accumulated in a time-dependent manner (24–48 h) in HepG2 cells (Figure 4B,C) and Huh7 cells (Figure 6B,C).

### 3.3. EGCG and Que Have No Influence on QSOX1 Activity

**Figure 7:**
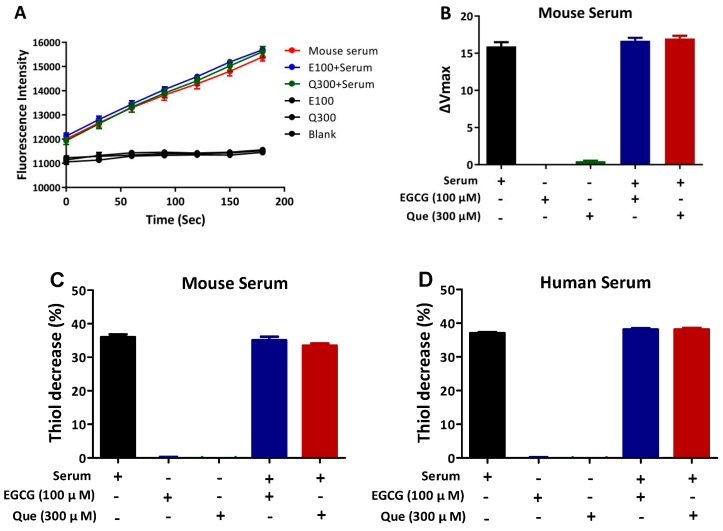
Influence of EGCG or Que on QSOX1 activity. EGCG (100 μM) or Que (300 μM) was incubated with serum at 37 °C for 2 or 3 h. (**A**,**B**) QSOX1 activity of mouse serum (3 min incubation) assessed via fluorescence-based assay; (**C**) DOC activity of mouse serum (15 min incubation) assessed via colorimetric assay; (**D**) DOC activity of human serum (15 min incubation) assessed via colorimetric assay. Data are presented as mean ± SEM (n = 3).

The above results demonstrate that EGCG and Que can increase intracellular QSOX1 accumulation. Many polyphenols, including EGCG and Que, have been reported to inhibit certain enzymatic activities such as SARS-CoV2 main protease, matrix metalloproteinase, glucose-6-phosphate dehydrogenase, and tyrosinase [32,33,34,35]. It is therefore important to determine whether EGCG and Que inhibit the enzymatic activity of QSOX1, given that only enzyme-active QSOX1 in HCC cells is helpful for reducing the invasion and metastasis of HCC cells and sensitizing sorafenib to kill HCC cells [6]. Israel et al. developed a sensitive method for assessing QSOX1 activity by using Amplex UltraRed and fluorescence assays; accordingly, they found a strong QSOX1 activity in mouse serum [29]. We found that this protocol could not be used in the lysates of HCC cells to detect QSOX1 activity, so we utilized mouse serum to investigate the influence of EGCG and Que on QSOX1 activity. The results showed that 100 μM EGCG and 300 μM Que did not affect QSOX1 activity in mouse serum (Figure 7A,B). DOC is a surrogate method for QSOX1 activity [11,36], and 100 μM EGCG or 300 μM Que did not affect DOC in either mouse or human serum (Figure 7C,D). These results, obtained using very high levels of EGCG or Que, indicate that EGCG and Que do not inhibit QSOX1 activity. A distinct mechanism by which EGCG inactivates enzymatic activity is the conjugation of EGCG quinone and the thiol of cysteine residue in the active site of an enzyme, forming a quinoprotein [33]. Active-site cysteines exist in resting QSOX1 as disulfide pairs (e.g., C70–C73, C449–C452, and C509–C512) [37,38]; thus, there is no free Cys-SH in the active site of QSOX1 available to be conjugated by EGCG quinone or Que quinone, although they may modify C165-SH and C237-SH. However, it is known that C165 and C237 residues are not conserved and are therefore unlikely to play a catalytic role [38]. This may help explain why neither EGCG nor Que inactivates QSOX1 activity.

### 3.4. Sorafenib Synergizes with EGCG or Que to Promote Apoptosis in HepG2 Cells

**Figure 8:**
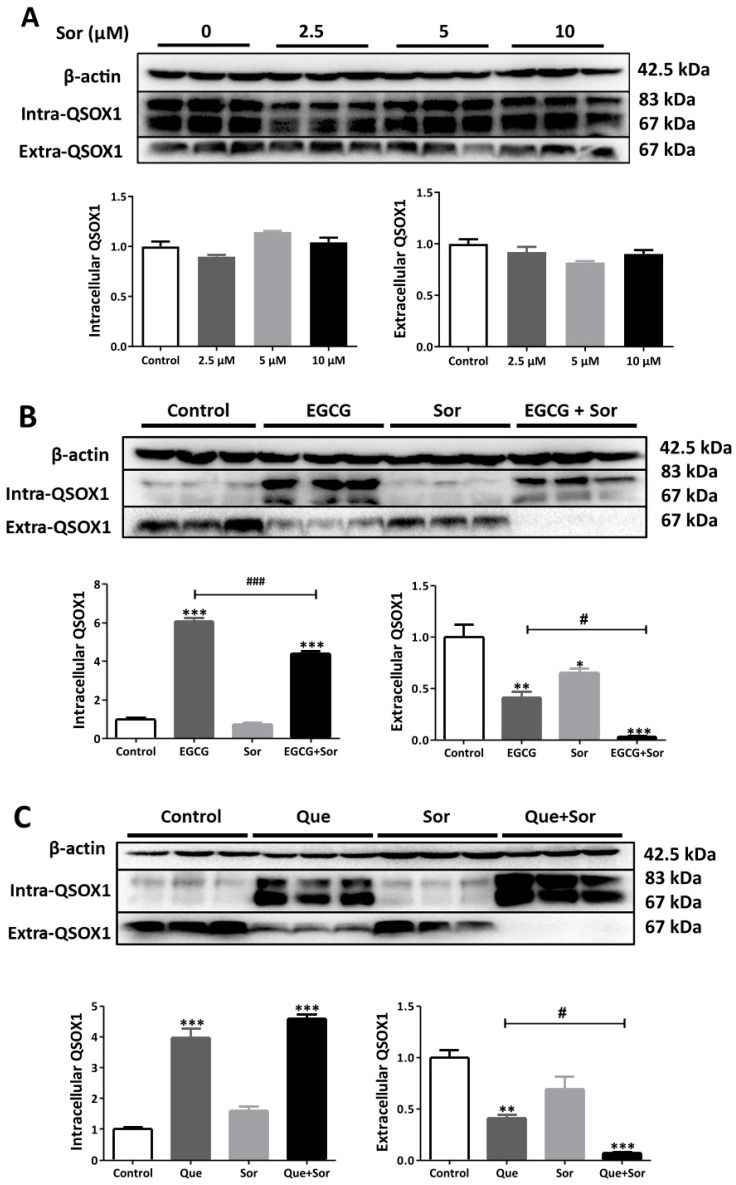
Influence of sorafenib (Sor) on QSOX1 in HepG2 cells. (**A**) Dose effect of sorafenib after 48 h of treatment; (**B**) influence of sorafenib (5 μM) plus EGCG (200 μM) on QSOX1 after 48 h of treatment; (**C**) influence of sorafenib (5 μM) plus Que (200 μM) on QSOX1 after 48 h of treatment. Data are presented as mean ± SEM (n = 3). Compared to the control, * *p* < 0.05, ** *p* < 0.01, and *** *p* < 0.001; compared to EGCG or Que, ^#^ *p* < 0.05 and ^###^ *p* < 0.001.

**Figure 9:**
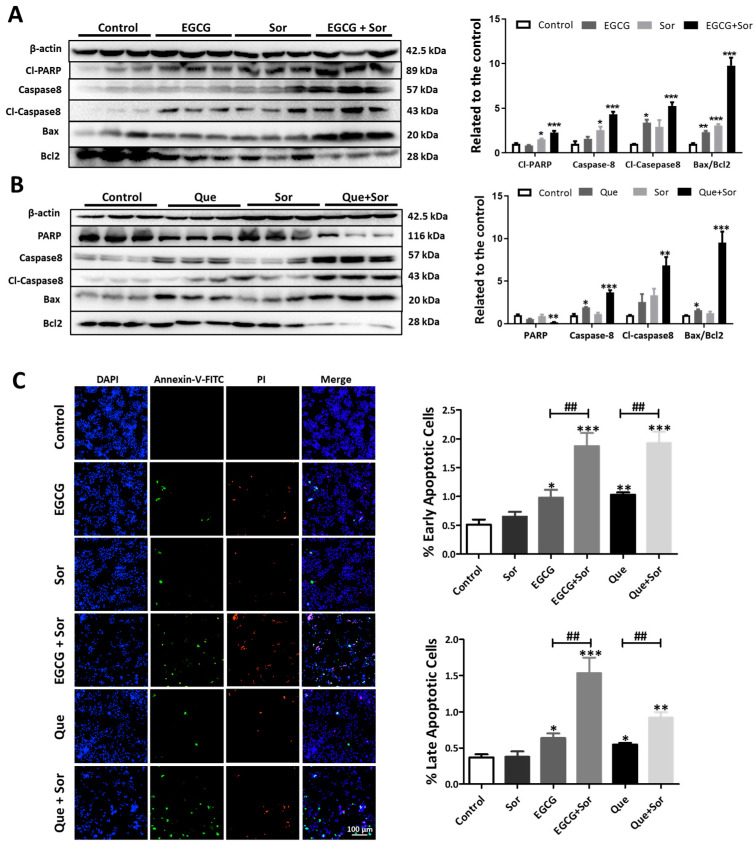
Influence of sorafenib (Sor), EGCG, Que, or their combinations on apoptosis in HepG2 cells. HepG2 cells were treated with EGCG (200 μM), Que (200 μM), sorafenib (5 μM), or their combination for 48 h. (**A**,**B**) Apoptosis-associated proteins; (**C**) early and late apoptosis, as indicated by high-content cell imaging. Data are presented as mean ± SEM (n = 3). Compared to the control, * *p* < 0.05, ** *p* < 0.01, and *** *p* < 0.001; compared to EGCG or Que, ^##^ *p* < 0.01.

It has been established that QSOX1-overexpressing HCC cell lines are more sensitive to sorafenib treatment [6]. We speculated that EGCG or Que might enhance sorafenib’s efficacy through inducing intracellular QSOX1 accumulation. The administration of sorafenib (5 μM) alone for 48 h had no prominent effect on cellular QSOX1 distribution in HepG2 cells (Figure 8A), but it substantially enhanced the EGCG- or Que-induced reduction in extracellular QSOX1 (Figure 8B,C). The combination of sorafenib (5 μM) and EGCG (200 μM) resulted in significantly increased intracellular QSOX1 and largely reduced extracellular QSOX1 as compared to the control (Figure 8B). The same was true for the combination of sorafenib (5 μM) and Que (200 μM) (Figure 8C). Thus, these HepG2 cells with QSOX1 distributions altered by EGCG or Que are more sensitive to 5 μM sorafenib. Indeed, apoptosis-associated proteins reached their maximum levels under the combined treatment of sorafenib and EGCG or Que (Figure 9A,B). Concerning the Bax/Bcl2 ratio (a pre-set rheostat within cells that predetermines the cell’s life or death [39,40]), while EGCG alone and sorafenib alone significantly increased the ratio 2.3-fold and 3.0-fold, respectively, their combination significantly raised the ratio 9.8-fold (Figure 9A). Meanwhile, the administration of Que alone only significantly increased the ratio 1.6-fold and sorafenib alone did not significantly alter the ratio, but their combination significantly raised the ratio 9.5-fold (Figure 9B). Evidently, the Bax/Bcl2 ratio in the combined treatments exhibited a synergistic increase profile compared to the single treatment. Moreover, either early apoptosis or late apoptosis, as reflected in a high-content cell imaging analysis, was at maximum levels under the combined treatment of sorafenib and EGCG or Que (Figure 9C). Concerning early apoptosis, while sorafenib alone did not significantly alter early apoptosis, EGCG alone or Que alone significantly increased its incidence by 2.0-fold and their combination with sorafenib significantly raised it 3.7-fold (Figure 9C). Concerning late apoptosis, sorafenib alone did not significantly alter late apoptosis, while EGCG or Que alone significantly increased late apoptosis 1.7-fold or 1.5-fold, respectively, and their combination with sorafenib significantly increased late apoptosis 4.2-fold or 2.5-fold, respectively (Figure 9C). These results have a ∆fa_xy_ value over zero, suggesting that sorafenib together with EGCG or Que can synergistically induce apoptosis in HepG2 cells; thus, EGCG and Que are potential candidates for sensitizing HCC cells to sorafenib.

### 3.5. Sorafenib Synergizes with EGCG or Que to Inhibit Migration and Invasion of Huh7 Cells

**Figure 10:**
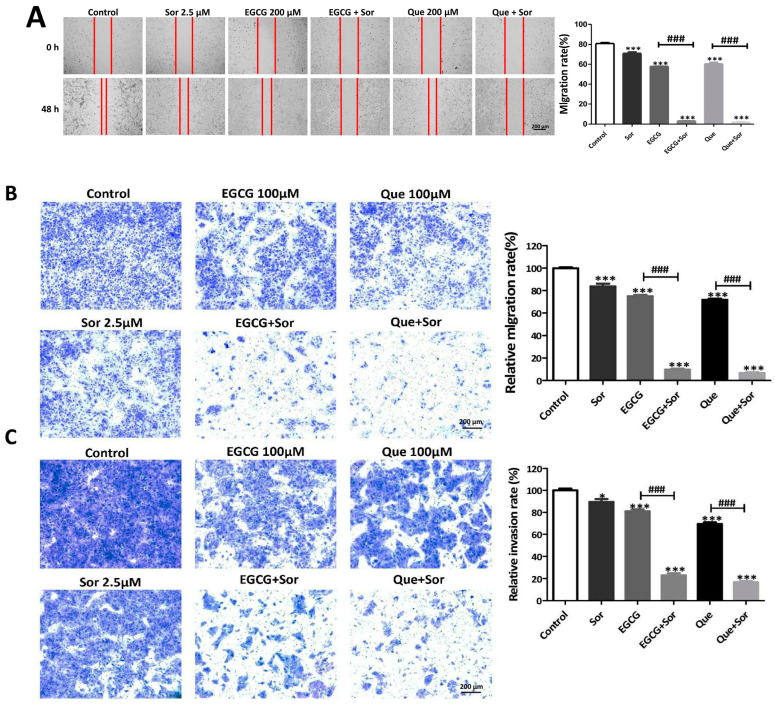
Influence of sorafenib (Sor), EGCG, Que, or their combination on migration and invasion of Huh7 cells. Huh7 cells were treated with EGCG, Que, sorafenib, or their combination for 48 h. (**A**) Migration of Huh7 cells treated with the drug at indicated concentrations for 48 h by wound healing assay; (**B**) effect of EGCG or Que in combination with Sor on Huh7 cell migration determined by Transwell assay; (**C**) effect of EGCG or Que in combination with Sor on Huh7 cell invasion determined by Transwell assay. Data are presented as mean ± SEM (n = 3). Compared to the control, * *p* < 0.05, *** *p* < 0.001; compared to the EGCG or Que, ^###^ *p* < 0.001.

Inspired by the synergistic effects discussed above, we further explored whether the combination treatment could synergistically inhibit the migration and invasion of HCC cells. In the wound healing migration assay, our preliminary experiments showed that the migration rate of HepG2 cells was relatively low (less than 50% after 48 h), whereas the migration rate of Huh7 cells was adequate (more than 80% after 48 h). Therefore, Huh7 cells are more suitable for characterizing how migration is influenced by the tested compounds. It was observed that 2.5 μM sorafenib alone and 200 μM EGCG alone significantly reduced the migration of Huh7 cells by 12% and 28%, respectively, but their combination synergistically inhibited the migration of Huh7 cells by 96% (∆fa_xy_ = 0.59) (Figure 10A). Moreover, 2.5 μM sorafenib alone and 200 μM Que alone significantly reduced the migration of Huh7 cells by 12% and 25%, respectively, but their combination caused a synergistic inhibitory effect on the migration of Huh7 cells by 98% (∆fa_xy_ = 0.63) (Figure 10A). Evaluating Huh7 cell migration via Transwell assay further supported the synergistic profile of sorafenib in combination with EGCG or Que. Specifically, 2.5 μM sorafenib alone and 100 μM EGCG alone significantly reduced migration by 16% and 25%, respectively, but their combination synergistically inhibited migration by 90% (∆fa_xy_ = 0.53) (Figure 10B). Moreover, 2.5 μM sorafenib alone and 100 μM Que alone significantly reduced migration by 16% and 28%, respectively, but their combination caused a synergistic inhibitory effect on migration of 93% (∆fa_xy_ = 0.53) (Figure 10B). A synergistically inhibitory effect of sorafenib in combination with EGCG or Que on Huh7 cell invasion could also be observed from the Transwell assay. Specifically, 2.5 μM sorafenib alone and 100 μM EGCG alone significantly reduced invasion by 11% and 19%, respectively, but their combination synergistically inhibited invasion by 77% (∆fa_xy_ = 0.50) (Figure 10C); meanwhile, 2.5 μM sorafenib alone and 100 μM Que alone significantly reduced invasion by 11% and 31%, respectively, while their combination had a synergistic inhibitory effect on invasion of 83% (∆fa_xy_ = 0.45) (Figure 10C).

## 4. Discussion

Accumulating evidence has revealed that intracellular QSOX1 acts as a tumor suppressor in HCC cells, whereas extracellular QSOX1 functions as a tumor promoter. Accordingly, a QSOX1 secretion inhibitor that increases intracellular QSOX1 and reduces extracellular QSOX1 could be used for HCC treatment. In this study, the influence of typical dietary polyphenols on the cellular distribution of QSOX1 was examined in human HCC cells. EGCG and Que were identified as QSOX1 secretion inhibitors, whereby the combination of sorafenib and EGCG or Que synergistically induced HCC cell apoptosis and inhibited HCC cell migration and metastasis.

As a multikinase inhibitor, sorafenib can suppress HCC cell proliferation and slow HCC progression [3]. Sorafenib appeared to have no significant impact on intracellular or extracellular QSOX1 levels (Figure 8A). EGCG or Que significantly increased intracellular QSOX1 and reduced extracellular QSOX1 in HCC cells (Figure 2). The overexpression of intracellular QSOX1 suppresses the Nrf2 pathway, thus sensitizing HCC cells to sorafenib [6]. Suppressing extracellular QSOX1 impairs cancer cell adhesion and growth [9,15,16]. Therefore, EGCG or Que would greatly sensitize HCC cells to sorafenib, allowing sorafenib to kill them more easily. Supporting this hypothesis, we have observed several synergistic events in terms of apoptosis induction in HCC cells when sorafenib was combined with EGCG or Que (Figure 9). A few studies have shown that sorafenib also inhibits HCC cell migration, invasion, and metastasis [41,42,43,44]. We observed the same phenomena here (Figure 10). Since extracellular QSOX1 is essential for establishing ECM which is pivotal for cancer cell migration, invasion, and metastasis [12,13,14], and because EGCG and Que are highly effective in reducing extracellular QSOX1, the inhibition of HCC cell migration, invasion, and metastasis by sorafenib is presumed to be enhanced in a QSOX1-deficient extracellular environment mediated by EGCG or Que. Supporting this hypothesis, we have observed a synergistic effect of sorafenib in combination with EGCG or Que on cell migration or cell invasion by using HCC cells (Figure 10).

It is important to address a complicating issue over certain dietary polyphenols; that is, the concentrations used in vitro are difficult to achieve in vivo, as indicated by plasma or serum concentrations. Nonetheless, these polyphenols at non-toxic doses in vivo could replicate the effects observed from in vitro experiments. Taking EGCG as an example, the effective EGCG concentration for inducing a significant change in biomolecules or biological pathways in cultured cells is normally at a range of 20–200 μM. However, the peak plasma EGCG concentrations following the intake of pharmacological doses of EGCG are 2–9 μM in mice and humans [45]. Despite this significant gap or discrepancy, pharmacological doses of EGCG or high-dose green tea could achieve a similar function in rodents to that identified in vitro using 20–200 μM EGCG. We present the following examples: (i) 80 μM EGCG suppressed HepG2 cell growth by inhibiting the IGF/IGF-1R axis [46], and similarly, the administration of 0.1% EGCG in the drinking water of mice (equivalent to 6 cups of green tea daily in humans) slowed hepatic tumor development by inhibiting the IGF/IGF-1R axis [47]; (ii) 120 μM EGCG promoted apoptosis in HCC cells by inhibiting NF-κB and PI3K/Akt [48], and similarly, liver cancer progression in rats was inhibited by drinking green tea rich in EGCG as it suppressed NF-κB and PI3K/Akt [49]; (iii) 200 μM EGCG inhibited Huh7 cell growth by suppressing the VEGF-VEGFR axis, and similarly, the administration of 0.1% EGCG in the drinking water of mice reduced the volume of Huh7 xenografts by inhibiting the VEGF-VEGFR axis [23]; (iv) 25–100 μM EGCG inhibited MMP2 and MMP9 in cultured cells, while the addition of 0.1% green tea polyphenols in drinking water significantly inhibited MMP2 and MMP9 in the prostate of TRAMP mice [50]; finally, (v) 50–200 μM EGCG inhibited the activation of primary mouse hepatic stellate cells by downregulating phospholipase C epsilon-1, and similarly, the oral administration of EGCG at a dose of 200 mg/kg/day (equivalent to 6 cups of green tea daily in humans) suppressed liver fibrosis in mice intoxicated with CCl4 by reducing phospholipase C epsilon-1 [51]. This significant discrepancy can be explained by plasma-ceruloplasmin-initiated rapid EGCG oxidation. EGCG readily undergoes auto-oxidation in neutral or alkaline pH conditions, resulting in the formation of oligomeric and polymeric compounds, collectively referred to as EGCG oxidation products [52,53]. Nonetheless, certain EGCG oxidation products still possess cytotoxic activity equivalent to EGCG in CaCo_2_ or TCA8113 cells [54]. It is widely recognized that transition metal copper dramatically promotes EGCG oxidation [55]. We have found that copper-containing ceruloplasmin promoted EGCG oxidation more effectively than copper based on the same molar copper. Such an important property of ceruloplasmin may help apprehend this significant discrepancy. Lower concentrations of EGCG detected in the plasma or serum may, in turn, imply that a high level of EGCG in the plasma or serum following a pharmacological dose of EGCG has been rapidly oxidized by plasma ceruloplasmin into large amounts of EGCG oxidation products with biological activities equivalent to EGCG. In this in vitro study, the effective EGCG concentrations were found in a range of 50–200 μM, as always. Based on the above analyses, we speculate that the observed effect of EGCG on QSOX1 in HCC cells could occur in HCC patients who consume around 6 cups of green tea daily, thereby enhancing the therapeutic effect of sorafenib. Our in vitro data also suggest that Que may enhance the therapeutic effect of sorafenib in vivo. This has been well demonstrated in a xenograft mouse model established using resistant Huh7 cells. Specifically, Que, sorafenib, and their combination, which did not increase toxicity compared to the single treatments, reduced tumor volume by 28%, 46%, and 87%, respectively, and reduced tumor mass by 22%, 42%, and 77%, respectively [24]. It can thus be anticipated that sorafenib would be more effective in HCC patients who consume adequate Que.

The limitation of the current study is that we cannot demonstrate why EGCG and Que inhibit QSOX1 secretion. It is known that the proteolytic cleavage of Golgi-localized QSOX1 is required for QSOX1 secretion [56]. EGCG and Que may act as the protease inhibitor to impair QSOX1 secretion from HCC cells. Since the exact identity of the protease catalyzing Golgi-resident QSOX1 remains to be determined, we cannot elucidate here whether EGCG or Que affects the protease responsible for the cleavage of Golgi-localized QSOX1. Future studies are needed to explore this potential mechanism, given the accumulating evidence showing that EGCG is effective at inhibiting multiple proteases. For example, EGCG is an inhibitor of metallo- and serine proteases such as MMP2 and MMP9 [50]. EGCG is also a potent inhibitor of leukocyte elastase with an IC50 as low as 0.4 μM [57]. EGCG can act as an inhibitor of urokinase [58]. The proteasome is a protease complex responsible for degrading most cellular proteins. EGCG exhibits proteasome-inhibitory activity [50]. EGCG, for example, inhibits the activity of SARS-CoV-2 main protease [59], with an IC50 of 0.26 μM, through preferentially conjugating with the thiol in active-site Cys145 to form a quinoprotein [33]. Other than the influence of Golgi-localized QSOX1 on proteolytic cleavage, EGCG or Que may impair QSOX1 secretion by disrupting the glycosylation modification of Golgi-localized QSOX1. Asparagine-linked glycosylation, commonly referred to as N-linked glycosylation, is the most common form of protein glycosylation and plays a pivotal role in proper protein folding, sorting, and trafficking. It has been demonstrated that a highly conserved N-linked glycosylation site in QSOX1 is essential for QSOX1 secretion from fibroblasts and other cell types [60]. EGCG and Que may disrupt the glycosylation modification of Golgi-localized QSOX1 by inhibiting the activities of oligosaccharyltransferase or α-glucosidases, which are indispensable for N-linked glycosylation [61,62]. Future studies examining the influence of EGCG and Que on endoplasmic reticulum oligosaccharyltransferase and α-glucosidases may be helpful in deciphering potential mechanisms by which EGCG and Que suppress QSOX1 secretion from HCC cells. Additional limitations of the present study include the lack of in vivo validation and patient-derived samples or clinical correlation studies, given that the present study primarily focuses on cellular mechanisms as opposed to systemic effects.

## 5. Conclusions

In summary, this study shows that dietary EGCG or Que effectively increases intracellular QSOX1, which functions as a tumor suppressor, and powerfully reduces extracellular QSOX1, which acts as a tumor promoter, by inhibiting QSOX1 secretion from HCC cells. Intriguingly, the combination of sorafenib and EGCG or Que exhibits multiple synergistic effects against HCC cells, including apoptosis induction and migration or invasion suppression. These synergistic effects strongly suggest that a potential sensitizing effect of HCC cells to sorafenib can be achieved by using EGCG or Que as an inhibitor of QSOX1 secretion from HCC cells, warranting further exploration.

## Data Availability

All data presented in this study are available on request from the corresponding authors. The data are not uploaded to a publicly accessible database.
